# Evaluating Sleep in Pediatric Cancer: A Scoping Review of Assessment Tools for Quality and Care

**DOI:** 10.1002/cam4.71051

**Published:** 2025-07-23

**Authors:** Elena Rostagno, Veronica Rivi, Pierfrancesco Sarti, Pietro Guastella, Dorella Scarponi, Johanna Maria Catharina Blom

**Affiliations:** ^1^ Pediatric Hematology and Oncology IRCCS Azienda Ospedaliero‐Universitaria di Bologna Bologna Italy; ^2^ Department of Biomedical, Metabolic, and Neural Sciences University of Modena and Reggio Emilia Modena Italy; ^3^ IRCCS Azienda Ospedaliero Universitaria di Bologna Bologna Italy; ^4^ Centre for Neuroscience and Neurotechnology University of Modena and Reggio Emilia Modena Italy

**Keywords:** actigraphy, pediatric oncology, sleep assessment, sleep disorders, sleep quality, wearables

## Abstract

**Background:**

Pediatric cancer patients experience unique and multifaceted sleep disturbances due to the disease, treatment regimens, and the hospital environment. These disruptions can detrimentally impact neurocognitive functioning, emotional well‐being, and overall quality of life, making accurate sleep assessment critical yet challenging in this population.

**Objective:**

To examine and evaluate the current tools used to assess sleep quality in pediatric oncology patients, with a focus on their reliability, feasibility, and relevance to clinical and research settings.

**Methods:**

A scoping review methodology was employed to identify and synthesize studies using various sleep assessment tools in pediatric cancer populations. Tools reviewed included actigraphy, sleep diaries, validated sleep scales, and polysomnography. Studies were analyzed for general reliability, feasibility in clinical and research contexts, and applicability to pediatric oncology‐specific concerns.

**Results:**

The review found that while actigraphy and sleep diaries are frequently used because of their noninvasive nature and relative ease of implementation, limitations exist in terms of consistency and interpretability. Sleep scales varied in their psychometric properties and relevance across age groups and treatment phases. Polysomnography, though considered the gold standard, was less feasible in routine clinical settings because of its complexity and cost. Across tools, variability was observed in the alignment between measured parameters and clinically relevant sleep issues in pediatric cancer patients.

**Conclusion:**

A wide range of tools exists for assessing sleep in pediatric oncology, each with distinct strengths and limitations. Selection of the most appropriate tool should consider the specific sleep concern, patient age, clinical context, and resource availability. This review provides a framework for clinicians and researchers to make informed choices, encouraging thoughtful integration of sleep assessments into both practice and study design.

## 1 Introduction

Healthy and restorative sleep is essential for the physical, emotional, and cognitive development of children and adolescents [1, 2, 3, 4]. Unfortunately, sleep‐related issues are widespread in this age group [3, 5, 6, 7], ranging from diagnosable *sleep disorders* [8, 9, 10]—such as insomnia, sleep apnea, and parasomnias, which meet specific clinical criteria, often require specialized treatment, and are linked to significant health impairments [11, 12, 13, 14, 15]—to transient *sleep disturbances*, which involve temporary disruptions in normal sleep patterns, like difficulty falling asleep or frequent awakenings, and may or may not suggest an underlying disorder [3, 12, 16, 17]. Additionally, there are more general *sleep problems*, a broad category that includes both mild, short‐term issues and chronic conditions that affect sleep quality and duration [6, 18, 19, 20].

Children with cancer are particularly vulnerable to sleep disruptions due to a combination of physical side effects from treatment, psychological stressors such as anxiety, and environmental barriers in hospital settings [11, 13, 21, 22, 23]. These disruptions manifest as difficulty falling asleep, excessive daytime sleepiness, and fragmented sleep patterns, compounding the physical and emotional toll of their illness [13]. The consequences of poor sleep in pediatric oncology patients profoundly affect emotional regulation, cognitive performance, and physical health [7, 24, 25]. Even after completing cancer treatment, many survivors experience persistent sleep disturbances or develop chronic sleep disorders [26], underscoring the importance of early identification and intervention to optimize both short‐ and long‐term outcomes [18, 26].

Despite the well‐documented impact of sleep problems in pediatric oncology, there is no standardized approach for assessing sleep in this population [17, 18, 24, 25]. Many studies report the measures they use without systematically evaluating their reliability, feasibility, and clinical applicability. Furthermore, a critical gap exists in how sleep assessment tools are selected: while some are chosen based on methodological rigor, others may be selected based on convenience, cost, or availability rather than their true suitability for pediatric cancer patients.

This review aims to provide a structured and systematic evaluation of sleep assessment tools used in pediatric oncology. To enhance clarity, we first examine the general reliability, feasibility, and clinical applicability of common sleep measurement paradigms—such as polysomnography, actigraphy, and subjective questionnaires—before discussing their specific use in pediatric oncology. We also critically evaluate why specific measures were chosen in past studies and whether they effectively captured the intended sleep constructs.

By offering a structured evaluation of sleep assessment tools and identifying best practices, this review provides a foundation for improving sleep research and clinical care in pediatric oncology. Our ultimate goal is to enhance the well‐being and long‐term quality of life for children and adolescents affected by cancer by ensuring that sleep assessments are reliable, feasible, and clinically meaningful.

## 2 Methods

### 2.1 PRISMA‐ScR Guidelines and Research Questions

This scoping review followed the methodology outlined by Arksey and O'Malley (2005) [27], consisting of five key steps: (1) identifying the research question, (2) identifying relevant studies, (3) selecting pertinent studies, (4) charting the data, and (5) collating, summarizing, and reporting the results. To ensure rigor and transparency in the conduct and reporting of the review, the Preferred Reporting Items for Systematic Reviews and Meta‐Analyses Extension for Scoping Reviews (PRISMA‐ScR) guidelines were adhered to [28]. A collaborative brainstorming session was held with the entire research team to refine the central research question: “In children and adolescents (aged 0–18) diagnosed with or survivors of cancer, what are the available sleep assessment tools, questionnaires, or instruments used to measure sleep quality, and how reliable and valid are these tools, regardless of a direct comparison group?”. This group discussion was instrumental in ensuring that the question addressed both the clinical needs and research gaps within the field.

### 2.2 PICO Methodology

The PICO (Population, Intervention, Comparison, Outcome) framework was employed to formulate the research question systematically [29, 30]. The key components of the PICO framework are as follows: Population (P): Children and adolescents (aged 0–18) diagnosed with cancer; Intervention (I): Sleep assessment tools, questionnaires, or instruments used to measure sleep quality; Comparison (C): No direct comparison was made; the focus of the review was to identify and evaluate available sleep assessment tools; Outcome (O): The primary outcome was the identification of reliable and valid tools or instruments used to measure sleep quality in pediatric cancer patients. Secondary outcomes included evaluating the strengths and limitations of these tools.

### 2.3 Identification of Relevant Studies, Study Selection, Data Charting, and Reporting the Results

A comprehensive literature search was performed across multiple databases, including PubMed, Web of Science, Cochrane Library, and PsycINFO, covering studies from January 2006 to July 2024. Only studies published in English were included. The search focused on studies that assessed or reported sleep quality, patterns, or disturbances in pediatric cancer patients, specifically children and adolescents diagnosed with cancer or neoplasm, regardless of cancer type. Eligible studies included original research articles, observational studies, clinical trials, and cohort studies that utilized validated tools or instruments designed to measure sleep quality. Studies involving patients at any stage of cancer treatment (e.g., pre‐treatment, ongoing treatment, post‐treatment, or palliative care) were included. The keyword selection was carefully crafted to capture sleep quality measurement in children and adolescents with cancer (ages 0–18). Keywords such as “tool OR questionnaire OR instrument,” “sleep quality,” “child* OR adolescent*,” and “cancer OR neoplasm” were used to identify relevant records. The initial search yielded 4884 records. To refine the results, additional filters specific to age and population were applied, narrowing the search to 2537 records. Further keyword refinement using logical operators reduced the search results to 2537 records. A citation analysis based on pivotal publications related to sleep quality measurement tools in pediatric oncology was also conducted, which identified an additional 3247 records.

**FIGURE 1:**
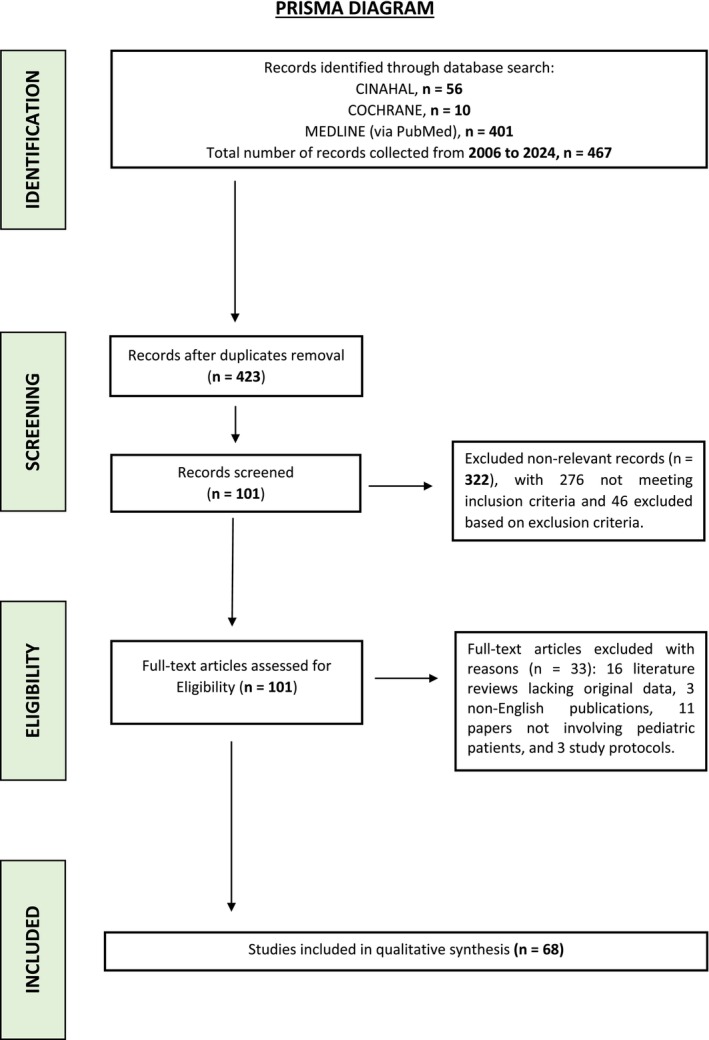
PRISMA diagram.

Exclusion criteria included systematic reviews, meta‐analyses, literature reviews, and theoretical papers. Studies focusing on mixed‐age populations where pediatric data were not separately analyzed, as well as studies that did not primarily focus on sleep quality or used sleep assessment as a secondary outcome, were excluded. Studies that did not employ recognized or validated sleep quality measurement tools and single‐case studies or anecdotal reports lacking generalizable data were also excluded. All study selection and data collection processes adhered to the PRISMA‐ScR guidelines (Figure 1) [28].

### 2.4 Data Extraction

Data extraction was conducted by two independent reviewers (E.R. and D.S.) and included information such as author(s), year of publication, study design, population characteristics (age, cancer type, therapy status), sample size, study setting, and the specific tool or instrument used to measure sleep quality. Any discrepancies in data extraction were resolved through discussion, and a third reviewer was consulted if needed. The extracted data were organized based on the type of sleep quality measurement tool, instrument, or questionnaire used in the included studies. Key findings from the selected studies were synthesized to assess the strengths and limitations of each tool. This synthesis provided a comprehensive overview of the applicability and effectiveness of these tools in pediatric oncology research (P.S. and V.R.).

## 3 Results

### 3.1 Study Selection and Methodology

A comprehensive literature search was conducted across multiple databases to identify studies addressing sleep quality in pediatric cancer patients. This search initially yielded 467 records. After the removal of duplicates, 423 articles remained for title and abstract screening. Of these, 322 were excluded for not meeting the predefined inclusion criteria, and an additional 46 were removed based on specific exclusion criteria, such as being non‐research articles or unrelated to the study's focus. The full‐text assessment phase involved 101 articles, of which 33 were excluded for the following reasons: 16 were literature reviews without original data; 3 were non‐English articles that could not be assessed due to language barriers; 11 did not involve pediatric populations, and 3 were study protocols rather than completed research.

This screening process resulted in the inclusion of 68 studies in the qualitative analysis (Figure 1). These studies, published between January 2006 and July 2024, are detailed in Table 1. The diversity of assessment tools underscores the complexity of evaluating sleep quality in pediatric cancer patients and the need for a multi‐dimensional approach. Additionally, the characteristics of each scale/questionnaire, including the dimensions and scoring, are reported in Table 2, whereas the reliability, validity, and usability of each tool, together with their strengths and limitations, are reported in Table 3. The following sections summarize the tools, questionnaires, and instruments used to assess sleep quality in these studies, highlighting their advantages, limitations, and variations in methodology.

**TABLE 1:** Studies included in this scoping review.

References	Study design	Participants	Pathology	In/off therapy	Setting	Tool, questionnaire, instrument
Akdeniz et al. [7]	Cross‐sectional	139 adolescents (13–18 years old)	Cancer	In/Off	In/Out patients	Sleep Assessment Scale for Children with Cancer—Adolescent Form
Abulhamail et al. [12]	Cross‐sectional	150 children and adolescents (2–18 years old)	Sickle cell disease	In/Off	In/Out patients	Pediatric Sleep Questionnaire
Burke et al. [31]	Cross‐sectional	132 parents (children 2–10 years old)	Cancer	In/Off	Out patients	Children's Sleep Habits Questionnaire Children's Oncology Child Adjustment Scale
Cheung et al. [21]	Cross‐sectional	70 survivors (8–25 years old)	ALL	Off	Out patients	Children's Sleep Habits Questionnaire Adolescent Sleep Habits Questionnaire Sleep average number of hours slept on a weekday, frequent delayed sleep onset (> 20 min), nighttime awakenings and premature awakenings
Cheung et al. [32]	Cross‐sectional	116 AYA (15–39 years old)	Sarcoma	In/Off	Out patients	Self‐reported average number of hours slept every day Sleep–Rest Fatigue subscale of the Pediatric Quality of Life Inventory Multidimensional Fatigue Scale
Daniel et al. [33]	Longitudinal	81 parents (children 3–12 years old)	ALL	In	Out patients	Sleep diary Abbreviated Children's Sleep Habits Questionnaire
Daniel et al. [6]	Case–control	167 survivors (16–30 years old)	Cancer	Off	Out patients	Pittsburg Sleep Quality Index
Daniel et al. [4]	This study utilizes baseline data from a pilot randomized controlled trial	61 AYA (12–25 years old)	Cancer	Off	Out patients	Pittsburgh Sleep Quality Index
Daniel et al. [34]	Cross‐sectional	45 children (8–17 years old) 102 caregivers (children 5–17 years old)	Cancer	In	Out patients	PROMIS Pediatric Sleep Scales
Daniel et al. [35]	Longitudinal	50 children/adolescents (8–21 years old)	Cancer	In	In/Out patients	Actigraph
Erickson et al. [36]	Prospective descriptive and correlational study	20 adolescents (12–19 years old)	Cancer	In	In/Out patients	General Sleep Disturbance Scale
Fisher et al. [37]^(p20)^	Data are part of a larger, longitudinal study	103 mothers 82 children (5–17 years old)	Cancer	In/Off	Out patients	Sleep duration
Fortmann et al. [38]	Cross‐sectional	202 AYA (13–24 years old)	Cancer	Off	Out patients	Pittsburgh Sleep Quality Index
Fortmann et al. [39]	Cross‐sectional	221 AYA (13–24 years old)	Cancer	In/Off	Out patients	Pittsburgh Sleep Quality Index
Gedaly‐Duff et al. [24]	Prospective and descriptive	9 children (8–16 years old) 6 fathers 7 mothers	ALL	In	Out patients	Sleep diary Actigraphy
Gordijn et al. [40]	Cross‐sectional	43 survivors (mean age 10.6 years old)	ALL	Off	Out patients	Children's Sleep Habits Questionnaire Adolescents Sleep Habits Questionnaire
Gordijn et al. [41]	Cross‐sectional	62 survivors (5–17 years old)	ALL	Off	Out patients	Children's Sleep Habits Questionnaire Adolescent Sleep Habits Questionnaire
Graef [5]	Cross‐sectional	76 survivors (mean age 17.84 years) 38 parents	Leukemia, aplastic anemia	Off	Out patients	Epworth Sleepiness Scale
Graef et al. [16]		37 children and adolescents (4–19 years old)	Medulloblastoma	In	In patients	Actigraph
Ho et al. [42]	Cross‐sectional	402 survivors (6–18 years old) 50 children with cancer	Cancer	In/Off	Out patients	Pittsburgh Sleep Quality Index
Hockenberry et al. [43]	Prospective, descriptive, within‐group, before and‐after	67 children and adolescents (7–18 years old)	Cancer	In	In patients	Actigraph
Hooke et al. [44]	Repeated measures	13 children and adolescents (10–18 years old)	Cancer	Off	Out patients	Adolescent Sleep–Wake Scale
Jacob et al. [25]	Descriptive	49 children and adolescents (8–17 years old)	Cancer	In	In patients	Numerical Rating Scale
Jacobs et al. [1]	RCT	34 children and AYA (12–21 years old)	Cancer	In	In patients	Actigraph Sleep diary
Ju et al. [45]	Cross‐sectional	54 adolescents (13–18 years old)	Osteosarcoma	In	In patients	Pittsburgh Sleep Quality Index
Khoirunnisa et al. [46]	Quasi‐experimental	64 children and adolescents (8–18 years old)	Cancer	In	In patients	Sleep Disturbance Scale for Children
Kim et al. [18]	Cross‐sectional	80 parents (children 2–10 years old)	Cancer	In/Off	Out patients	Sleep Disturbance Scale for Children‐Disorders of Initiating and Maintaining Sleep Subscale Child Sleep Hygiene Scale
Klages et al. [19]	Longitudinal	84 children and adolescents (8–20 years old)	Cancer	In/Off	In/Out patients	Multiple Sleep Latency Test Polysomnography
Kudubeş et al. [47]	Cross‐sectional	140 adolescents (13–18 years old)	Cancer	In/Off	Out patients	Pittsburgh Sleep Quality Index
Linder et al. [14]	Exploratory, descriptive, multiple‐case study	15 children (mean age 8.8 years old)	Cancer	In	In patients	Actigraph
Lubas et al. [48]	Randomized double‐blind placebo‐controlled trial	580 cancer survivors (mean age: 33.5 years old; 26 years post‐diagnosis)	Cancer	Off	Out patients	Pittsburgh Sleep Quality Index Actigraph
Lubas et al. [10]	Cohort study	477 cancer survivors (mean age: 34.3, 25.4 years from diagnosis)	Cancer	Off	Out patients	Pittsburgh Sleep Quality Index Actigraph
MacArtney et al. [49]	Cross‐sectional	50 survivors (2–18 years old)	Brain tumors	Off	Out patients	Multi Symptoms Assessment Scale
Malboeuf‐Hurtubise et al. [26]	Prospective quasi‐experimental pretest–posttest	14 adolescents (11–18 years old)	Cancer	In/Off	Out patients	Pittsburgh Sleep Quality Index
Merz et al. [50]	Case–control	99 children/adolescents (8–18 years old)	Cancer	Off	Out patients	Actigraph PedsQL Multidimensional Fatigue Scale
Niel et al. [51]	Cross‐sectional	78 children and adolescents (6–20 years old)	Craniopharyngioma	In	In patients	Multiple Sleep Latency Test Polysomnography
Nunes et al. [52]	Prospective	118 children and adolescents (8–18 years old)	Cancer	In	In patients	Actigraph
Orsey et al. [53]	Cross‐sectional	36 children and adolescents (8–18 years old)	Cancer	In	In patients	Actigraph Sleep diaries Visual Analog Scale
Padmanabhan et al. [54]	Cross‐sectional	105 children/adolescents (8–17 years old)	Cancer	In/Off	Out patients	Ultra‐short Munich Chronotype Questionnaire
Peersmann et al. [11]	Cross‐sectional	565 adolescents (12–26 years old)	Cancer	In/Off	Out patients	
Pickering et al. [9]	Cross‐sectional	61 children and adolescents (0–18 years old)	Brain tumors	In/Off	In patients	Polysomnography Multiple Sleep Latency Test Pediatric Daytime Sleepiness Scale Children's Sleep Habits Questionnaire
Pickering et al. [55]	Cross‐sectional	68 children and adolescents (0–18 years old)	Brain tumors	In/Off	Out patients	Sleep diaries Actigraph
Pierzynski et al. [56]	Qualitative interview study	51 survivors (8–17 years old) 35 caregivers	Cancer	Off	Out patients	Interviews
Pilotto et al. [8]	Case–control	29 children and adolescents (2–16 years old)	Brain tumors	Off	Out patients	Child's Sleep Habits Questionnaire
Rodgers et al. [57]	Repeated‐measures	327 children and adolescents (3–18 years old)	ALL	In	In/Out patients	Children's Sleep–Wake Scale Adolescent Sleep–Wake Scale
Rogers et al. [58]	Prospective (second analysis)	82 children and adolescents (5–18 years old)	ALL	In	Out patients	Actigraph
Ruble et al. [59]	Descriptive	62 survivors (8–18 years old)	Cancer	Off	Out patients	Pediatric Sleep Questionnaire‐Sleep Disordered Breathing Subscale
Russell et al. [3]	Case–control	45 survivors (8–18 years old)	ALL	Off	Out patient	Sleep diary Actigraph Children's Sleep Habits Questionnaire
Setoyama et al. [2]	Cross‐sectional	11 children (2–12 years old)	Cancer	In	In patients	Actigraph Sleep diary
Sriasih et al. [60]	Quasi‐experimental pre‐post test	58 children and adolescents (7–18 years old)	Cancer	In	In patients	Sleep Problem in Children Scale
Steur et al. [20]	Observational, longitudinal	124 children (median age 5.1 years old)	ALL	In	Out patients	Actigraph Children's Sleep Habits Questionnaire Adolescent Sleep Habits Questionnaire Sleep Self‐Report
Su et al. [61]	Longitudinal	18 children and adolescents (3–19 years old)	Cancer	In	Out patients	Children's Sleep Quality Scale
Szymczak et al. [62]	Qualitative interviews	32 children and AYA (8–21 years old)	AML	In	In/Out patients	Interviews
Traube et al. [63]	Cross‐sectional	56 children and adolescents (0–18 years old)	Cancer	In	In patients	Actigraph Brief Infant Sleep Questionnaire Children's Sleep Habits Questionnaire Sleep at Memorial Sloan Kettering Cancer Center Questionnaire
Vallance et al. [64]	Retrospective	88 children and adolescents (5–18 years old)	ALL	In	Out patients	Actigraph
van Litsenburg et al. [65]	Descriptive	17 children and adolescents (2–18 years old)	ALL	In	Out patients	Children's Sleep Habits Questionnaire
van Hulst et al. [66]	Double‐blind, randomized controlled trial	52 children and adolescents (3–18 years old)	ALL	In	Out patients	Actigraph Sleep diary Sleep Disturbance Scale for Children
van Hulst et al. [67]	Prospective study	105 children (median age: 5.4 years old)	Cancer	In	Out patients	Sleep Disturbance Scale for Children
van Schaik [65]	Case reports	4 cases (12–16 years old)	Cancer	Off	Out patients	Polysomnography Epworth Sleepiness Scale Multiple Sleep Latency Test
Verberne et al. [15]	Cross‐sectional	31 children and adolescents (4–18 years old)	Cancer	Off	Out patients	Sleep Disorder Scale for Children Epworth Sleepiness Scale
Walker et al. [68]	Descriptive, longitudinal	51 children and adolescents (10–19 years old)	Cancer	In	In patients	Sleep Routines “Before You Were Sick” Questionnaire Sleep diary Adolescent Sleep Wake Scale Adolescent Sleep Hygiene Scale
Walker et al. [13]	Descriptive, longitudinal	38 children and adolescents (10–19 years old)	Cancer	In	Out patients	Actigraph Sleep diary
Williamson Lewis et al. [69]	Cohort	579 survivors (13–17 years old)	Cancer	Off	Out patients	Multi Symptoms Assessment Scale
Wolfe et al. [70]	Cohort	104 children (7–12 years old)	Cancer	Off	Out patients	PediQUEST‐Memorial Symptom Assessment Scale
Wu et al. (2019) [71]	Cross‐sectional	100 children and adolescents (13–18 years old)	Cancer	In/Off	Out patients	Pittsburgh Sleep Quality Index
Zhou et al. [72]	Longitudinal	10 AYA (15–40 years old)	Cancer	Off	Out patients	Sleep logs diaries Pittsburgh Sleep Quality Index Insomnia Severity Index
Zhou et al. [73]	Cross‐sectional	22 AYA (15–25 years old)	Cancer	Off	Out patients	Pediatric Daytime Sleepiness Scale Sleep diaries Insomnia Severity Index
Zupanec et al. [74]	Pilot randomized controlled trial	20 children (4–10 years old)	ALL	In	Out patients	Actigraph Sleep diary Children's Sleep Habits Questionnaire

Abbreviations: ALL, acute lymphoblastic leukemia; AML, acute myeloid leukemia; AYA, adolescents and young adults; PROMIS, patient reported outcomes measurement information system.

**TABLE 2:** Characteristics of the scale/questionnaire.

Scale, questionnaire	N. items	Version	Target	Time interval of assessment	Dimensions	Scoring
Adolescent Sleep Habits Questionnaire (ASHQ) [75]	54‐item 50‐item	Proxy Self	13–17 years old > 12 years	1 week	Bedtime behavior and sleep onset, sleep duration, sleep anxiety, night waking, morning waking, and daytime sleepiness	Items were rated on a 4‐point scale. Some items of both questionnaires were reversed to consistently make a higher score indicative of more disturbed sleep
Adolescent Sleep Hygiene Scale (ASHS) [76]	28‐items	Self	11–18 years old	1 month/1 week	Physiological, cognitive, emotional, sleep environment, substances, and sleep stability	6‐point scale (never, once in a while, sometimes, quite often, frequently not always, and always). The total score can be obtained by summing all items with higher scores indicating better sleep hygiene behaviors
Adolescent Sleep Wake Scale (ASWS) [77]	28‐item	Self	12–18 years old	1 week	Going to bed, falling asleep, maintaining sleep, going back to sleep, and returning to wakefulness	6‐point scale (never, once in a while, sometimes, quite often, frequently if not always, always). Higher scores indicate better sleep quality
Baylor Infant Sleep Questionnaire (BISQ) [78]	13‐item	Proxy	0–3 years old	1 week	Sleep‐onset time, nocturnal sleep duration, daytime sleep duration, total sleep duration, night wakings, and nocturnal wakefulness	Clinical interpretation
Children's Sleep Habits Questionnaire (CSHQ) [79]	45‐item 35‐item 33‐item 26‐item (self)	Proxy Self	4–12 years old 8–12 years old	1 week	Bedtime behavior and sleep onset; sleep duration; anxiety around sleep; behavior occurring during sleep and night wakings; sleep‐disordered breathing; parasomnias; and morning waking/daytime sleepiness	Items are rated on a 3‐point scale: “usually” if the sleep behavior occurred five to seven times/week; “sometimes” for two to four times/week; and “rarely” for zero to one time/week. Some items were reversed to consistently make a higher score indicative of more disturbed sleep. Total scores range from 33 to 99 and higher total scores indicated greater sleep disturbance. A total score above 41 suggested a sleep disorder
Children's Sleep Hygiene Scale (CSHS) [80]	24‐items	Proxy	2–10 years old	1 week	Cognitive, physiological, emotional, environmental bedtime routine, and sleep stability	6‐point scale (never, once in a while, sometimes, quite often, frequently‐if not always, and always). The total score can be obtained by summing all items with higher scores indicating better sleep hygiene
Children's Sleep Quality Scale (CSQS) [81]	10‐item + a face rating scale (from 1—very poor sleep to 6—very good sleep)	Self/Proxy	3–19 years old	1 week	Sleeping difficulties	Each item was rated from 0 (*none*) to 4 (*every day*). The total score is derived from the score of the child's subjective feeling of sleep (after reverse scoring) plus the score for sleeping difficulty, and could range from 1 to 46. A higher score indicates poorer sleep quality
Children's Sleep Wake Scale (CSWS) [82]	28‐item 28‐item	Proxy Self	3–6 years old 7–12 years old	1 week	Going to bed, falling asleep, maintaining sleep, going back to sleep, and returning to wakefulness	6‐point scale (never, once in a while, sometimes, quite often, frequently if not always, always). Higher scores indicate better sleep quality
Epworth Sleepiness Scale (ESS) [83]	8‐item	Proxy Self	4 years old 5–18 years old		Daytime sleepiness	Total scores range from 0 to 24, with higher scores indicating greater sleepiness
General Sleep Disturbance Scale (GSDS) [84]	21‐item	Self	> 12 years	1 week	Sleep quality, daytime function, sleep aids	8‐point Likert scale for frequency of 0 (*not at all*) to 7 (*every day*); mean score > 3 is criteria for possible sleep disorder using DSM‐IV criteria
Insomnia Severity Index (ISI) [85]	7‐item	Self	> 15 years	2 weeks	The perceived severity of insomnia symptoms (initial, middle, terminal), the degree of satisfaction with sleep, interference with daytime functioning, noticeability of impairment, and concern caused by sleep problems	5‐point Likert scale. A total score of 8 and above is associated with subthreshold insomnia, while a total score of 15 or above is associated with clinical insomnia
Pediatric Daytime Sleepiness Scale (PDSS) [86]	8‐item	Self	11–15 years old		Daily sleep patterns, school achievement, mood, sleepiness, quality of life, and extracurricular activities	4‐point Likert scale. Total score varying from 0 to 32. Higher scores indicated greater levels of sleepiness
PROMIS Sleep Disturbance Scale (PROMIS SD) [87]	8‐item (SD) 8‐item (SRI)	Proxy Self	5–17 years old 8–17 years old	1 week	SD: problems with falling and staying asleep and sleep quality. SRI: children's daytime sleepiness, difficulties waking up, and the impact of sleepiness on thinking, mood, behavior, and daily activities	Items were rated on a scale of “never” (0) to “always” (5), with higher scores indicating more problematic sleep
Pediatric Sleep Questionnaire (PSQ) [88]	22‐item	Proxy	2–18 years old	1 month	Sleepiness snoring, in‐attention/hyperactivity	Responses are “yes” = 1, “no” = 0, and “don't know” = missing. More than 8 positive responses may indicate a problem with sleep‐related breathing disorder
Pittsburgh Sleep Quality Index (PSQI) [89]	19‐item (The other five questions are to be answered by the spouse or a roommate and not included in the scoring)	Self	≥ 10 years old	1 month	Subjective sleep quality, sleep latency (the length of time it takes to fall asleep), sleep duration, habitual sleep efficiency (the ratio of the total time spent asleep per night compared with the total amount of time spent in bed), sleep disturbance, use of sleeping medications, and daytime dysfunction	Each component was scored on a scale that ranged from 0 (*no difficulty*) to 3 (*severe difficulty*). The seven component scores are summed to produce a global score ranging from 0 to 21, with greater scores indicating poorer sleep quality. The cut‐off score is set at five, to identify cases with clinical sleep disorders.
Sleep Disturbance Scale for Children (SDSC) [90]	26‐items	Proxy	2–18 years old	1 week	Disorders of initiating and maintaining sleep; sleep breathing disorders; disorders of arousal/nightmares; sleep–wake transition disorders; disorders of excessive somnolence; and sleep hyperhidrosis	5‐point scale. The total score ranges between 26 and 130. Higher scores indicate more severe symptoms

Abbreviations: ASHQ, Adolescent Sleep Habits Questionnaire; ASHS, Adolescent Sleep Hygiene Scale; ASWS, Adolescent Sleep Wake Scale; BISQ, Brief Infant Sleep Questionnaire; CSHQ, Children's Sleep Habits Questionnaire; CSHS, Children's Sleep Hygiene Scale; CSQS, Children's Sleep Quality Scale; CSWS, Children's Sleep Wake Scale; ESS, Epworth Sleepiness Scale; GSDS, General Sleep Disturbance Scale; ISI, Insomnia Severity Index; PDSS, Pediatric Daytime Sleepiness Scale; PROMIS SD SRI, Patient Reported Outcome Measurement Information System Sleep Disturbance Sleep‐Related Impairment; PSQ, Pediatric Sleep Questionnaire; PSQI, Pittsburgh Sleep Quality Index; SDSC, Sleep Disturbance Scale for Children.

**TABLE 3:** Comparison of sleep assessment tools for pediatric oncology patients: emphasizing reliability, validity, and usability.

Assessment tool	Reliability	Validity	Usability	Strengths	Limitations
Polysomnography (PSG)	High inter‐rater reliability for sleep staging and event detection due to standardized scoring methods	Valid for diagnosing a wide range of sleep disorders (e.g., sleep apnea, periodic limb movement disorder) and assessing sleep architecture	Requires overnight stay in a controlled lab setting; resource‐intensive; specialized equipment and trained personnel required	Gold standard for evaluating sleep disorders and sleep architecture; detects physiological disturbances in sleep (e.g., apnea, fragmented sleep)	Limited feasibility for routine or longitudinal monitoring; not practical for outpatient or home settings due to complex setup and need for technician supervision; disruptive for children and families because of multiple sensors worn overnight; data quality in unattended (Type II) systems may be compromised by artifacts and lack of supervision; Type III systems lack EEG, preventing sleep staging and limiting diagnostic scope to mainly respiratory disorders
Actigraphy	Reliable for tracking rest‐activity patterns, particularly over extended periods	Moderately valid for estimating sleep duration, efficiency, and timing; less valid for detecting sleep stages or specific disorders like apnea	Highly usable: non‐invasive, wrist‐worn, and portable; suitable for at‐home or outpatient monitoring; cost‐effective compared to PSG	Practical for routine assessments; provides long‐term sleep data in real‐world settings; ideal for monitoring treatment‐related changes in sleep patterns	Limited diagnostic capability—unable to measure sleep stages or detect detailed physiological changes; prone to overestimating sleep in individuals with minimal movement during wakefulness; may misclassify quiet wakefulness as sleep; less accurate in detecting sleep fragmentation or brief awakenings; cannot diagnose specific sleep disorders such as apnea or periodic limb movements; data quality and interpretation depend on device placement and algorithms used
Sleep diaries	Reliability depends on consistency in reporting and adherence to logging routines	Provides a valid subjective snapshot of sleep habits when used consistently over time	Easy to use; requires minimal training; can be completed by children (with assistance) or caregivers	Captures sleep patterns over time; inexpensive and convenient for home use; complements objective tools like actigraphy	Susceptible to recall bias and inaccuracies; relies on patient or caregiver perception, which may not align with actual sleep behaviors; requires consistent daily completion, which may be burdensome over time; limited in capturing night‐time awakenings or exact sleep onset/offset times without objective corroboration
Children's Sleep Habits Questionnaire (CSHQ)	Reliable when completed by parents who consistently observe the child's sleep behaviors	Valid for assessing common pediatric sleep disturbances, including bedtime resistance, sleep anxiety, and parasomnias	Parent‐reported; easy to administer for ages 4–10; widely used in research and clinical settings	Tailored to younger children's sleep issues; captures a broad spectrum of behaviors relevant to pediatric oncology (e.g., night wakings, anxiety)	Dependent on parental perception, which may not reflect the child's actual sleep experience; may overlook subtle or physiological sleep issues such as sleep apnea or circadian rhythm disturbances; limited by subjective reporting and potential response bias
Adolescent Sleep Habits Questionnaire (ASHQ)	Moderately reliable, as adolescents may underreport or inaccurately recall sleep patterns	Valid for assessing adolescent‐specific sleep issues like circadian rhythm shifts, delayed sleep phase, and daytime sleepiness	Self‐reported; designed for ages 11–18; requires basic literacy and understanding of sleep‐related concepts	Adolescent‐focused; captures age‐specific challenges such as social and biological influences on sleep (e.g., circadian misalignment)	Limited ability to detect physiological sleep disorders; relies on adolescent self‐report, which may be influenced by underreporting, misinterpretation, or social desirability bias; retrospective format may reduce accuracy; limited sensitivity to short‐term changes or night‐to‐night variability
Adolescent Sleep Hygiene Scale (ASHS)	Reliable for assessing sleep hygiene behaviors in adolescents	Valid for evaluating behaviors affecting sleep quality and duration	Self‐reported; designed for ages 12–18	Provides insight into sleep habits and hygiene practices	Does not measure physiological sleep disorders or provide objective data; limited to self‐reported behaviors, which may be influenced by recall bias or social desirability; not designed to detect changes in sleep architecture or diagnose clinical sleep conditions
Adolescent Sleep Wake Scale (ASWS)	Reliable for assessing sleep–wake patterns in adolescents	Valid for measuring sleep disturbances related to circadian rhythm and sleep onset difficulties	Self‐reported; designed for adolescents; requires comprehension of sleep habits	Focuses on adolescent‐specific sleep behaviors and disturbances	Limited in detecting physiological sleep disorders such as sleep apnea; relies on adolescent self‐report, which may be influenced by recall bias, comprehension issues, or social desirability; does not measure environmental or contextual sleep factors
Baylor Infant Sleep Questionnaire (BISQ)	High reliability when completed consistently by parents	Valid for assessing infant sleep patterns, night wakings, and sleep duration	Parent‐reported; easy to use for infants and toddlers	Designed specifically for infants; captures sleep issues in early childhood	Based on parental report, which may be biased or inaccurate; lacks objective data; limited capacity to differentiate between normative and clinically significant sleep variations in infancy; less useful beyond toddler age
Children's Sleep Wake Scale (CSWS)	Reliable for evaluating children's sleep–wake behaviors	Valid for assessing sleep disturbances across different sleep dimensions	Parent‐reported; simple format for use in clinical and research settings	Provides detailed information on children's sleep habits	Dependent on parental observation, which may not reflect true sleep behaviors; lacks physiological or objective measurement; may not detect transient or subtle changes in sleep patterns
Children's Sleep Hygiene Scale (CSHS)	Reliable for assessing sleep hygiene in children	Valid for evaluating the impact of sleep hygiene practices on sleep quality	Parent‐reported; suitable for ages 6–12	Helps identify areas for improving bedtime routines	Focuses on behavioral aspects only; does not diagnose clinical sleep disorders or measure sleep outcomes directly; parent‐reported format may introduce bias or misunderstanding of sleep hygiene constructs
Children's Sleep Quality Scale (CSQS)	Reliable for assessing subjective sleep quality in children	Valid for measuring perceived sleep quality and disturbances	Parent‐ or self‐reported; easy to administer	Provides insight into a child's perception of sleep health	Relies on subjective impressions; lacks physiological or objective validation; may not differentiate between specific sleep problems or comorbid issues such as anxiety
Epworth Sleepiness Scale (ESS)	High reliability in assessing daytime sleepiness	Valid for detecting excessive daytime sleepiness and its impact	Short and easy to administer; self‐reported	Widely used in clinical and research settings; helps identify sleep disorders	Designed for adults, though used in adolescents; does not account for age‐specific sleep patterns or causes of sleepiness; not diagnostic; lacks information on nighttime sleep structure
General Sleep Disturbance Scale (GSDS)	Reliable for evaluating sleep disturbances in adults	Valid for assessing various sleep issues, including insomnia and poor sleep quality	Self‐reported; used in different populations	Covers multiple sleep disturbance dimensions	Broad, self‐reported assessment; does not differentiate between types of sleep disorders; limited utility in identifying root causes; lacks alignment with diagnostic criteria or objective validation
Insomnia Severity Index (ISI)	High reliability for assessing insomnia severity	Valid for diagnosing and monitoring treatment outcomes in insomnia	Quick and simple self‐report questionnaire	Useful in clinical practice for evaluating sleep interventions	Subject to self‐report bias; not suitable for identifying comorbid sleep disorders or distinguishing between acute and chronic insomnia; does not capture physiological data or sleep architecture
Pediatric Daytime Sleepiness Scale (PDSS)	Reliable for assessing daytime sleepiness in children	Valid for evaluating sleep‐related daytime impairment	Self‐reported; suitable for school‐aged children	Identifies sleepiness‐related academic and behavioral concerns	Focuses only on daytime symptoms; does not evaluate sleep duration, nighttime awakenings, or specific sleep disorders; adolescent self‐report may be inconsistent
PROMIS Sleep Disturbance Scale (PROMIS SD)	Reliable for measuring sleep disturbances across populations	Valid for assessing subjective sleep difficulties	Self‐reported; used in clinical and research settings	Standardized assessment with broad applicability	Provides a general measure of sleep disturbance but lacks specificity; not diagnostic; does not assess physiological aspects of sleep; may be influenced by mood or health status
Pediatric Sleep Questionnaire (PSQ)	Reliable for identifying pediatric sleep disorders	Valid for assessing obstructive sleep apnea and other sleep disturbances	Parent‐reported; easy to complete	Useful in clinical screening for sleep‐disordered breathing	Strong for identifying sleep‐disordered breathing, but limited for non‐respiratory disorders (e.g., insomnia, restless legs); parent‐reported format may miss adolescent‐specific issues; does not include objective measures
Pittsburgh Sleep Quality Index (PSQI)	High reliability for assessing sleep quality in adults	Valid for measuring subjective sleep quality and disturbances	Self‐reported; widely used in research and clinical settings	Comprehensive sleep assessment tool	Designed for adults, though used in various age groups; not validated for all pediatric populations; lacks objective sleep data; does not specify causes of poor sleep quality
Sleep Disturbance Scale for Children (SDSC)	Reliable for evaluating sleep disorders in children	Valid for identifying insomnia, parasomnias, and sleep‐disordered breathing	Parent‐reported; designed for pediatric populations	Useful for clinical assessment of pediatric sleep disturbances	Lacks objective or physiological sleep data; parent report may not capture adolescent‐specific sleep problems or internal experiences

*Note:* An overview of various sleep assessment tools used in pediatric oncology, focusing on their reliability, validity, usability, strengths, and main limitations. Objective tools like PSG and actigraphy offer high reliability and detailed validity but may be more resource intensive. Subjective tools, such as questionnaires and diaries, are easier to implement in outpatient settings but rely heavily on individual consistency. To avoid redundancy, only limitations that specifically differentiate each tool from others are listed here; common limitations shared across similar tools are acknowledged but not repeated for each individual instrument. This approach allows clinicians to better appreciate the unique considerations of each tool while supporting a comprehensive assessment of pediatric oncology patients' sleep health.

### 3.2 Actigraphy

Actigraphy is a non‐invasive method that measures sleep–wake patterns using a wrist‐worn accelerometer to track limb movements. It provides an objective measure of sleep duration and quality, allowing for continuous assessment of sleep–wake cycles. Actigraphy has become a primary evaluation tool for circadian rhythm disorders, as it can effectively monitor disruptions in sleep patterns, which are often key indicators of these disorders. This capability is supported by recent studies that highlight its utility in identifying circadian misalignments and other sleep disturbances associated with various conditions. In the studies reviewed, actigraphy was employed in 16 studies [1, 2, 3, 5, 9, 20, 24, 43, 49, 53, 58, 63, 64, 68, 74, 91], either as a standalone tool [5, 14, 43, 52, 64] or in combination with sleep diaries and/or questionnaires [3, 14, 24, 43, 52].

Key sleep variables assessed included total sleep time, sleep onset latency, wake after sleep onset, and the number of awakenings. Actigraphy offers significant advantages, such as continuous monitoring and an objective measure of sleep–wake activity, providing insights into circadian rhythm patterns [4, 19, 48, 67, 92, 93] and helping detect irregularities that other tools may miss. However, there are some limitations to its use. Actigraphy is sensitive to movement artifacts, which can lead to the misclassification of sleep states, particularly in cases where limb movements are unrelated to actual sleep. Additionally, there are inconsistencies in scoring algorithms and epoch lengths across studies [10, 19, 48, 67, 92], which can introduce variability in the data. In some studies, actigraphs were typically worn on the non‐dominant wrist [3, 9, 14, 24, 43], though others recommended using the dominant wrist for improved accuracy [58].

Assessment durations varied, ranging from 3 days [14, 24, 42, 52, 63] to 14 days [9], with studies such as Hockenberry et al. [43] employing actigraphy during therapy and for 48 h post‐discharge, while Pickering et al. [9] conducted assessments over a 2‐week period. These variations in recording duration and methodology can impact the consistency and comparability of results across studies. Further inconsistencies emerged in epoch length settings, ranging from 30 s [3, 9, 58, 68] to 1 min [1, 2, 5, 14, 43, 74], and in data processing methods. Some studies used zero‐crossing mode [14, 63], which counts the number of times the signal crosses zero, while others relied on digital integration [9, 94], tracking activity counts above a threshold to indicate wake states. These methodological differences underscore the flexibility of actigraphy but also pose challenges in standardizing data interpretation, especially when evaluating circadian rhythms or comparing results across different populations. Despite these challenges, actigraphy remains a valuable tool for evaluating sleep–wake activity and circadian rhythm disturbances [95], particularly in pediatric populations where objective monitoring is necessary for understanding complex sleep patterns.

### 3.3 Sleep Diaries

Sleep diaries provide self‐reported data on sleep patterns, offering insight into subjective sleep experiences and disturbances. Twelve studies incorporated sleep diaries, typically recording bedtime, wake‐up time, sleep onset latency, night awakenings, and daytime naps [1, 2, 3, 13, 24, 33, 53, 55, 68, 72, 74]. The duration of diary use varied, ranging from 3 [2, 24] to 34 days [33, 72], with an average duration of 5–7 days [3, 13, 53, 68, 74]. Participants generally completed the diaries themselves [2, 3, 13, 72], though seven studies [1, 20, 24, 33, 53, 93, 96] involved parental supervision or direct parental reporting. While sleep diaries offer valuable insights into sleep behaviors, their accuracy depends on consistent adherence and honest reporting. Additionally, variability in diary content and recall biases can influence data reliability.

### 3.4 Scales and Questionnaires

Various self‐report and proxy‐report instruments have been employed to assess sleep quality in pediatric populations, each providing valuable insights while also presenting certain strengths and limitations. These tools, typically used in combination, aim to capture the complex nature of sleep disturbances, particularly in vulnerable groups such as pediatric cancer patients, where both treatment‐related factors and psychological stressors can impact sleep. One of the most widely used tools is the Pittsburgh Sleep Quality Index (PSQI) [89], which has been utilized in 10 studies [4, 6, 26, 38, 39, 42, 45, 47, 71, 91]. The PSQI is a validated and comprehensive questionnaire that assesses overall sleep quality over a defined period, providing a broad overview of sleep disturbances. However, its reliance on subjective reporting and variations in recall periods can limit its consistency and accuracy. The tool asks participants to reflect on their sleep behaviors over a 1‐month period, which can lead to recall bias, especially in populations such as pediatric cancer patients who may experience irregular sleep patterns due to treatments, anxiety, or other factors. Additionally, the subjective nature of the responses introduces variability, which can affect the tool's ability to capture nuanced sleep disturbances. The Children's Sleep Habits Questionnaire (CSHQ [79]), used in five studies [11, 20, 21, 40, 41] and in conjunction with other instruments in seven additional studies [3, 9, 20, 31, 33, 63, 74], is another prominent tool in the assessment of pediatric sleep. The CSHQ provides detailed information on a range of sleep behaviors, including bedtime routines, sleep onset, night awakenings, and daytime sleepiness. It is often used in pediatric populations over the age of eight, with parents typically completing the survey. While the CSHQ offers valuable insights, its application varies across studies, and its reliance on parental reports can introduce biases, especially in younger children where the parent's observations may not fully capture the child's subjective experience. Furthermore, the scale's ability to assess the impact of more complex sleep disorders, such as those arising from cancer treatments, may be limited by its general focus on sleep habits and behaviors.

The Adolescent Sleep Habits Questionnaire (ASHQ [97]), which has been used in four studies [21, 40, 41, 61], specifically targets adolescents, providing insight into the sleep patterns and behaviors typical of this age group. While it is a useful tool for assessing sleep in adolescents, its applicability to younger children is limited. The ASHQ, like the CSHQ, is susceptible to the same biases related to parental reporting, particularly for children under the age of 12, who may not be able to self‐report on their sleep experiences. Additionally, because it focuses on adolescent sleep patterns, it may not fully address the unique sleep‐related challenges faced by younger pediatric patients. The Epworth Sleepiness Scale (ESS [83]), used in three studies [5, 15, 65], measures daytime sleepiness, which is a key indicator of disrupted sleep. While it provides valuable information on how sleepiness affects daytime functioning, it lacks comprehensive data on overall sleep quality. This limitation makes it less useful for understanding the full scope of sleep disturbances in pediatric cancer patients, who may experience fragmented sleep due to treatments, pain, or psychological distress. The ESS is also limited by its reliance on self‐report, which may be influenced by the participant's awareness or perception of their sleepiness. The Children's Sleep Wake Scale (CSWS [82]) and Adolescent Sleep Wake Scale (ASWS [77]) assess sleep–wake patterns during cancer treatment and off‐therapy periods [57, 68], providing valuable information on how treatment schedules, side effects, and other factors may influence sleep. However, variability in scoring methods and interpretation across studies can impact consistency, reducing the tool's reliability [7, 11, 32, 54, 66, 93] in longitudinal studies or studies with diverse populations. Both scales are also limited by the reliance on parental reporting or proxy reports, which may not capture the full complexity of sleep–wake patterns in children undergoing cancer treatment.

The Sleep Disturbance Scale for Children (SDSC [90]), used in three studies [15, 46, 98], is another important tool, particularly for identifying specific sleep disturbances such as night terrors, sleepwalking, and sleep apnea. However, the SDSC is often used in conjunction with other scales to provide a more complete picture of sleep quality. While it offers valuable information on specific sleep disturbances, it does not fully address the broader patterns of sleep quality or capture the impact of other sleep factors like insomnia or daytime sleepiness. Other instruments that have been employed in sleep assessments include the Children Sleep Hygiene Scale (CSHS [80]), Insomnia Severity Index (ISI [85]), Pediatric Daytime Sleepiness Scale (PDSS [86]), Children's Sleep Quality Scale (CSQS [76]), and Brief Infant Sleep Questionnaire (BISQ [78]). Each of these tools targets specific aspects of sleep, such as sleep hygiene, insomnia severity, daytime sleepiness, and overall sleep quality. For instance, CSHS evaluates habits and routines that may influence sleep quality, such as irregular sleep schedules and caffeine consumption. While the SHS provides insight into lifestyle factors affecting sleep, it may not capture the impact of deeper sleep disorders, such as those related to cancer treatments. The PDSS assesses how daytime sleepiness affects functioning and cognitive‐behavioral performance, but it too relies on subjective reports, which can be influenced by the participant's self‐awareness or willingness to accurately report sleepiness. The ISI [11], which quantifies insomnia severity, can struggle to distinguish primary insomnia from other sleep disorders, making it difficult to pinpoint the underlying cause of sleep disturbances in pediatric cancer patients. The CSQS and BISQ are designed to assess sleep onset, maintenance, and night‐waking in children and infants, though they may not fully capture the nuances of sleep disorders across diverse age groups. One consistent limitation across many of these tools is the reliance on parent‐reported data, which may introduce bias. Parents' perceptions of their child's sleep may not always align with the child's subjective experience, particularly in younger children who may not be able to articulate their sleep disturbances. Furthermore, many of these tools do not address the complexities of sleep disorders that may arise specifically from cancer treatments, such as the effects of chemotherapy, radiation, or the emotional toll of a cancer diagnosis. Thus, while various self‐report and proxy‐report instruments provide useful insights into sleep quality, they each have their limitations. The reliance on subjective reports, recall bias, and variability in application across studies all contribute to inconsistencies in the data. Despite these limitations, these tools remain invaluable in the ongoing effort to understand and improve sleep quality in pediatric cancer patients, and they emphasize the importance of using a multi‐faceted approach to assess sleep disturbances comprehensively. Combining multiple assessment tools can help mitigate some of these challenges, providing a more accurate and holistic understanding of pediatric sleep quality.

### 3.5 Other Instruments

Beyond traditional sleep assessment tools, some studies employed alternative methodologies to enhance the understanding of sleep‐related issues. Polysomnography (PSG) was used in four studies [11, 20, 60, 93]. Traditionally, PSG has been conducted in specialized sleep labs, where patients are monitored overnight in a controlled environment. This method, while comprehensive, often presents significant challenges for pediatric cancer patients, who are already navigating the physical and emotional stress of their illness and treatment. The prospect of spending additional nights in a hospital or sleep clinic can exacerbate anxiety, potentially skewing sleep patterns and making it more difficult to capture accurate sleep data. Moreover, the logistics of transporting children to and from sleep labs can be burdensome for families, leading to a scenario where the very process meant to assess and improve sleep quality becomes an additional source of stress. However, recent advancements in technology have introduced a more practical solution: Type II and Type III unattended PSG systems that can be used at home [99]. These systems allow for sleep monitoring outside the confines of sleep labs, offering a more comfortable and natural environment for patients [99]. Type II PSG systems are capable of providing comprehensive data, measuring everything from brain activity to heart rate, while Type III systems, though less extensive, still capture key parameters such as respiratory patterns and oxygen levels [99]. Both systems enable clinicians to monitor sleep over multiple nights, providing a more detailed and accurate picture of a patient's sleep quality without the need for a hospital stay. The shift to at‐home monitoring offers a host of benefits, particularly for pediatric cancer patients [99]. First and foremost, it reduces the stress of unfamiliar hospital environments, allowing children to sleep in their own beds. This not only eases emotional distress but may also result in more typical sleep patterns, providing a clearer and more accurate representation of the patient's sleep behavior. Families also find it easier to manage the logistics of at‐home monitoring. Instead of navigating hospital schedules or making multiple trips to a sleep clinic, parents can simply set up the equipment in the comfort of their home, making it a more convenient and less disruptive process for both parents and children. Another key advantage is the potential for cost savings. Traditional sleep studies in labs can be expensive, with costs rising significantly when multiple nights of monitoring are required. By using unattended PSG systems at home, these costs can be reduced, benefiting both healthcare systems and families. Additionally, the ability to monitor sleep over several consecutive nights allows clinicians to gather more reliable data on sleep patterns, giving them a better understanding of the patient's sleep issues [99].

The flexibility of at‐home monitoring enables a more comprehensive evaluation, which is crucial for pediatric cancer patients whose sleep may fluctuate due to treatments, medications, and the emotional toll of their illness. However, there are some challenges that come with the shift to home‐based monitoring. Type III systems, for example, while offering essential insights into sleep disturbances such as apnea, do not measure all of the parameters that in‐lab PSG does. For instance, they do not capture brain activity, which can limit the ability to diagnose certain sleep disorders. Furthermore, while the devices are designed to be user‐friendly, there is still the possibility of technical issues, such as poor electrode placement or device malfunction, which could lead to inaccurate data. Unlike in a sleep lab, where technicians can immediately troubleshoot any problems, home‐based systems rely on the patient or caregiver to set up the equipment correctly, which can sometimes lead to errors. Additionally, while at‐home systems can collect valuable data, they lack the immediate oversight of a professional technician who can adjust settings or assess the quality of data in real time. This means that any issues with the equipment or data collection process might only be identified after the fact, potentially delaying diagnosis or treatment. Despite these limitations, the introduction of Type II and Type III unattended PSG systems represents a significant step forward in sleep research and patient care, especially for pediatric cancer patients. The ability to monitor sleep at home alleviates many of the logistical, emotional, and financial barriers associated with traditional sleep studies. As this technology continues to evolve, it holds the promise of making sleep monitoring more accessible, comfortable, and accurate, ultimately leading to better outcomes for children facing the dual challenges of sleep disturbances and cancer treatment.

Overall, the studies reviewed highlight a wide range of methodologies used to assess sleep quality in pediatric oncology patients, each with unique strengths and limitations. Actigraphy provides objective, continuous monitoring but varies in scoring methods and sensitivity to movement artifacts. Sleep diaries offer valuable subjective insights but are prone to recall bias and adherence issues. Questionnaires and scales allow for structured assessments but differ in scope, reliability, and applicability across age groups. Given these variations, a multi‐method approach is necessary to comprehensively assess sleep quality in pediatric cancer patients. By improving the reliability and comparability of sleep assessments, clinicians and researchers can better identify and address sleep disturbances in this vulnerable population, ultimately enhancing patient care and long‐term well‐being.

## 4 Discussion

### 4.1 Complexity and Variability of Sleep Disturbances in Pediatric Oncology

Despite notable progress in understanding sleep disturbances in pediatric oncology, the complexities of addressing these issues remain considerable. Sleep disturbances in pediatric cancer patients are multifactorial, influenced by a combination of the disease itself, treatment regimens [43, 51, 64], and individual patient factors. The current literature reveals significant methodological inconsistencies and gaps in the sleep assessment tools available, limiting their effectiveness and broad applicability in clinical settings. One of the most prominent limitations in the research is the diversity of sleep disturbances experienced by pediatric oncology patients. These disturbances vary widely depending on factors such as the type of cancer, phase of treatment, and individual characteristics like age and comorbidities. For instance, children undergoing chemotherapy may experience sleep disruptions due to nausea or pain, whereas survivors may suffer from persistent insomnia or fatigue. This variability complicates the generalization of findings across the entire pediatric oncology population, and it hinders the development of standardized tools or interventions that could be universally applied.

### 4.2 Environmental and Symptom Overlap

Additionally, sleep disturbances are often compounded by environmental factors, such as the disruption of normal sleep–wake patterns during hospital stays, early‐morning treatments, and the psychological stress associated with the illness [1, 2, 11, 43, 59, 60]. However, many studies fail to account adequately for these factors, which play a critical role in evaluating sleep health in pediatric oncology patients. Additionally, the overlap of sleep disturbances with other symptoms—like pain, anxiety, and fatigue [1, 2]—further complicates the task of isolating sleep disturbances as a distinct concern.

### 4.3 Impact on Families and Quality of Life

Not least, the impact of sleep disturbances extends beyond the individual patient, significantly affecting family dynamics. Parents and caregivers often experience increased stress and burden due to their child's sleep‐related issues, which can exacerbate the challenges of managing pediatric cancer care [22, 100]. Sleep issues such as irregular sleep schedules, reduced sleep duration, and poor sleep quality can also impair overall well‐being, affecting the patient's emotional, cognitive, and physical health [14, 23, 59, 101, 102]. Therefore, effective assessment must not only address the child's sleep but also consider the broader impact on family dynamics [56, 103].

### 4.4 Future Directions in Research and Practice

Looking ahead, future research in this area must prioritize several key directions:

1. *Refining Sleep Assessment Tools*: There is a need for more precise, context‐specific tools that can capture the full range of sleep disturbances [3, 59] seen in pediatric oncology. Traditional tools like actigraphy should be further evaluated for their reliability and feasibility in clinical practice [48, 104, 105, 106], especially in different cancer subgroups and stages of treatment. In particular, longitudinal data on sleep disturbances throughout the treatment continuum and into survivorship is crucial for developing more accurate assessments.
2. *Long‐Term Data Collection*: Longitudinal studies are needed to track sleep disturbances over time in pediatric oncology patients, from diagnosis through treatment and into post‐treatment survivorship. Such data would allow us to better understand the progression of sleep disturbances and identify critical periods when targeted interventions could be most beneficial.
3. *Development of Tailored Interventions*: As sleep disturbances in pediatric oncology are influenced by both medical and psychological factors, future research should focus on developing personalized interventions that address these multifactorial causes. These interventions should be adaptable to the patient's age, developmental stage, and specific treatment needs, and they should be validated for both efficacy and feasibility in clinical settings.
4. *Exploration of Emerging Technologies*: Digital technologies, such as wearable devices and AI‐driven platforms, hold promise for revolutionizing sleep assessment in pediatric oncology. However, their clinical reliability and applicability remain uncertain in pediatric oncology. Most wearables are validated in healthy populations, raising concerns about their accuracy in this specific group. Rigorous validation tailored to pediatric oncology is required. Integrating wearable data into electronic health records (EHRs) could also support real‐time clinical decision‐making, but challenges remain regarding infrastructure, data security, and health equity.
5. *Equity and Accessibility*: Finally, future research must consider the accessibility and equity issues surrounding wearable technologies and other sleep assessment tools. High costs, lack of reliable internet access, and cultural barriers can prevent underserved populations from benefiting from these technologies. Efforts to make these tools more accessible, such as through subsidized programs or shared devices, will be critical to ensure equitable access to sleep assessments for all pediatric oncology patients.

### 4.5 Limitations

We recognize that several limitations must be acknowledged. In particular, the variability in research methodologies—ranging from subjective questionnaires to objective measures like actigraphy and polysomnography—complicated the synthesis of data. These methodological differences may have introduced heterogeneity into the review's conclusions, which we have sought to address by discussing the strengths and limitations of each tool. Moreover, our search strategy, while extensive, may have overlooked relevant studies published in non‐English languages or studies with limited sample sizes, which could have provided additional insights into the topic. Additionally, the absence of longitudinal data, particularly in the context of cancer treatment phases and survivorship, is another significant gap in the literature that limits our ability to draw comprehensive conclusions.

## 5 Conclusion and Future Perspectives

This review has identified significant gaps in the current landscape of sleep assessment in pediatric oncology. Moving forward, it is essential to bridge these gaps by refining existing tools, developing new technologies, and focusing on long‐term, longitudinal research. Future studies should also consider the impact of sleep disturbances [12, 107] on families and the broader healthcare system, ensuring that interventions are designed to address the multifactorial nature of sleep issues in pediatric cancer patients.

Despite increasing attention to this topic, current methods are limited, as no single approach fully captures the spectrum of sleep issues in this population. The lack of longitudinal studies, tailored interventions, and caregiver support further exacerbates these challenges. Future research should prioritize the development of robust, individualized assessment tools that consider both clinical and psychosocial factors, alongside sustainable interventions that address diverse patient needs.

By addressing these challenges, we can move toward a more comprehensive and effective approach to assessing and managing sleep disturbances in pediatric oncology, ultimately improving the overall quality of life for both patients and their families.

Despite increasing attention to this topic, current methods fall short of capturing the full complexity of sleep disturbances in pediatric oncology. No single approach can adequately address the spectrum of issues affecting these patients. Tailored, multi‐method assessment strategies are needed to accommodate diverse clinical presentations and developmental stages. Future research should refine sleep assessment tools, incorporate longitudinal data, and develop targeted interventions that are age‐ and treatment‐specific. A significant gap exists in the evaluation of wearable devices for sleep monitoring: although promising for continuous and non‐invasive assessment, their clinical applicability remains uncertain, as they are mostly validated in healthy populations. Rigorous validation protocols specific to pediatric oncology are urgently needed. Integration of wearable and AI‐driven data into clinical workflows will also require overcoming infrastructure limitations, ensuring data security, and addressing health equity. Equitable implementation, supported by subsidized programs and co‐designed tools, is essential. By bridging these gaps and integrating personalized assessment tools and emerging technologies into care models, we can improve sleep health and the overall quality of life for pediatric oncology patients and their families.

## Author Contributions

**Elena Rostagno:** conceptualization (equal), data curation (equal), formal analysis (equal), validation (equal), visualization (equal), writing – original draft (equal). **Veronica Rivi:** conceptualization (equal), data curation (equal), investigation (equal), methodology (equal), validation (equal), visualization (equal), writing – original draft (equal). **Pierfrancesco Sarti:** data curation (equal), formal analysis (equal), software (equal), validation (equal), visualization (equal), writing – review and editing (equal). **Pietro Guastella:** data curation (equal), investigation (equal), writing – review and editing (equal). **Dorella Scarponi:** conceptualization (equal), data curation (equal), investigation (equal), resources (equal), supervision (equal), visualization (equal). **Johanna Maria Catharina Blom:** conceptualization (equal), funding acquisition (equal), project administration (equal), resources (equal), supervision (equal), visualization (equal), writing – review and editing (equal).

## Conflicts of Interest

The authors declare no conflicts of interest.

## Data Availability

Data will be made available upon reasonable request to the corresponding author.
